# Epistemic Injustice in Rheumatoid Arthritis Care: A Narrative Review of Invisible Suffering, Ageism, and Treatment Delay

**DOI:** 10.7759/cureus.103383

**Published:** 2026-02-10

**Authors:** Ryuichi Ohta, Kunihiro Ichinose

**Affiliations:** 1 Community Care, Unnan City Hospital, Unnan, JPN; 2 Rheumatology, Shimane University Faculty of Medicine, Izumo, JPN

**Keywords:** ageism, aging, diagnostic delay, epistemic, general medicine, health equity, justice, patient-reported outcome measures, rheumatoid arthritis

## Abstract

Despite advances in disease-modifying therapies and treat-to-target strategies, many patients with rheumatoid arthritis (RA) continue to experience persistent pain, fatigue, and functional impairment. These symptoms are particularly common among older adults and are frequently under-recognized in clinical practice. This study examines RA care through the lens of epistemic injustice to explore how patients’ experiential knowledge is interpreted, valued, or discounted, and how these processes contribute to treatment delay in aging societies. We conducted a narrative review of peer-reviewed literature addressing patient experiences, diagnostic and treatment delays, aging-related factors, and epistemic concepts relevant to RA care. Publications were identified through targeted database searches and citation tracking across rheumatology, social medicine, and medical ethics. Studies were examined conceptually to identify patterns of testimonial and hermeneutical injustice operating across the RA care continuum. Across the included literature, patients’ reports of pain, fatigue, and functional decline were frequently afforded reduced credibility when objective inflammatory markers appeared controlled, reflecting testimonial injustice. Hermeneutical injustice was evident when patients, particularly older and socially isolated individuals, lacked interpretive frameworks to recognize symptoms as pathological rather than age-related. These intersecting epistemic failures operated both before and after diagnosis, contributing to delayed help-seeking, delayed referral, and delayed treatment adjustment despite ongoing suffering. Treatment delay in RA cannot be fully explained by structural or biomedical factors alone. Epistemic injustice plays a critical role in shaping symptom interpretation and clinical decision-making, particularly in older adults. Addressing these epistemic dimensions by integrating patient testimony and patient-reported outcomes more meaningfully into care may promote timelier, more equitable, and more responsive RA management in aging populations. This review uniquely reframes treatment delay in RA as an epistemic problem, demonstrating how ageism and social isolation systematically distort symptom interpretation beyond structural or biomedical explanations.

## Introduction and background

Rheumatoid arthritis (RA) is a chronic inflammatory disease that significantly affects physical function, quality of life, and social participation [1]. Although advances in disease-modifying antirheumatic drugs (DMARDs) and the widespread adoption of treat-to-target strategies have improved inflammatory control, many patients continue to experience persistent pain, fatigue, and functional impairment despite apparently adequate disease activity management [2,3]. These residual symptoms are particularly common among older adults and are often insufficiently addressed within conventional biomedical models of RA care [4,5].

In aging societies, the clinical recognition and management of RA in older adults present distinct challenges [6]. Musculoskeletal pain, stiffness, and reduced mobility are frequently interpreted as inevitable consequences of aging rather than manifestations of treatable inflammatory disease [7]. Such age-based assumptions may delay symptom recognition, delay referral to rheumatology specialists, and ultimately delay the initiation of effective therapy [8]. Importantly, these delays cannot be fully explained by structural barriers to healthcare access alone. Rather, they reflect deeper problems related to how patients’ experiences are interpreted, validated, and translated into clinical action.

The concept of epistemic injustice, introduced by Fricker, offers a valuable lens for examining these issues [9]. Epistemic injustice refers to harms inflicted on individuals in their capacity as knowers. It is commonly described in two forms: testimonial injustice, in which a speaker’s account is afforded reduced credibility due to prejudice, and hermeneutical injustice, which arises when individuals lack shared interpretive resources to make sense of their experiences [9]. In healthcare settings, epistemic injustice has been increasingly discussed in relation to chronic illness and pain, where patients’ lived experiences may be discounted or rendered unintelligible within dominant biomedical frameworks [10].

RA represents a particularly salient case of epistemic injustice in clinical practice. Disease activity assessment relies heavily on laboratory markers and composite indices, while patients’ reports of pain, fatigue, and daily functional burden are often regarded as subjective or secondary [11]. This epistemic hierarchy can marginalize patient knowledge and obscure clinically meaningful suffering. Among older adults, ageism and social isolation further intensify these dynamics [12]. Social isolation may limit opportunities to compare symptoms or articulate concerns, while culturally entrenched narratives of “normal aging” deprive patients of the interpretive frameworks needed to recognize their symptoms as pathological [13]. These processes constitute hermeneutical injustice and can directly contribute to treatment delays.

Despite growing emphasis on patient-centered care and shared decision-making in rheumatology, the role of epistemic injustice in shaping diagnostic trajectories and treatment timing in RA remains underexplored [14]. This narrative review examines RA care through the lens of epistemic injustice, with particular attention to aging, ageism, and social isolation. By synthesizing literature from rheumatology, medical ethics, and social medicine, we seek to reframe treatment delay not only as a biomedical or logistical problem but also as an epistemic one, and to identify pathways toward more equitable and responsive RA care in aging societies. By applying the concept of epistemic injustice, this review makes visible how persistent suffering in RA, particularly among older adults, becomes structurally embedded in clinical reasoning and care pathways. While existing literature has emphasized structural barriers, access, and communication, and frameworks such as patient-centered care aim to improve dialogue and shared decision-making, these approaches do not fully explain why patients’ symptom reports may still be systematically discounted. Epistemic injustice provides additional explanatory power by highlighting how credibility deficits and interpretive gaps can devalue patients’ experiential knowledge, contributing to delayed recognition and treatment beyond issues of communication or access alone.

## Review

Methods

Study Design

This study was conducted as a narrative review to explore how epistemic injustice manifests in RA care, with particular attention to aging, ageism, social isolation, and treatment delay. A narrative approach was chosen to allow conceptual integration of heterogeneous literature spanning rheumatology, medical ethics, social medicine, and qualitative health research, which would not be amenable to formal systematic review methods.

Search Strategy

We conducted a targeted literature search using PubMed/MEDLINE (Medical Literature Analysis and Retrieval System Online) to identify relevant publications. The search was performed between January 2000 and December 2025 to capture contemporary developments in RA care and emerging applications of epistemic injustice in healthcare research. Searches were conducted iteratively across disciplines and refined through citation tracking. Key search terms included combinations of: “rheumatoid arthritis,” “patient experience,” “pain,” “fatigue,” “diagnostic delay,” “treatment delay,” “aging,” “older adults,” “ageism,” “social isolation,” “epistemic injustice,” “testimonial injustice,” “hermeneutical injustice,” and “patient-reported outcomes.”

Eligibility Criteria

For this narrative review, we included peer-reviewed articles published in English that addressed at least one of the following domains relevant to epistemic injustice in RA care: (i) patient experiences of RA, particularly pain, fatigue, or functional impairment; (ii) diagnostic or treatment delay in RA; (iii) age-related factors, including ageism or social isolation, in RA or chronic musculoskeletal disease; or (iv) theoretical or empirical discussions of epistemic injustice, patient knowledge, credibility, or related epistemic concepts in healthcare contexts.

Given the integrative and conceptual nature of this review, a broad range of study designs was considered eligible, including quantitative studies, qualitative research, mixed-methods studies, narrative reviews, conceptual papers, and ethical analyses. Conference abstracts, non-peer-reviewed commentaries, and studies focusing exclusively on pediatric populations were excluded to maintain relevance to adult RA care and ensure interpretive depth.

Data Extraction and Synthesis

Consistent with the narrative and conceptually driven design of this review, formal data extraction using predefined quantitative variables was not undertaken. Instead, included articles were examined for their conceptual relevance to epistemic injustice in RA care, encompassing both testimonial and hermeneutical forms. During the review process, we focused on how patients’ accounts of symptoms and illness experiences were interpreted, validated, or discounted within clinical encounters and broader social contexts.

Key themes were identified inductively through iterative reading, with particular attention to mechanisms through which testimonial injustice, such as credibility deficits assigned to patients’ narratives, and hermeneutical injustice, such as the absence of shared interpretive resources to make sense of symptoms, shape clinical reasoning and care trajectories. Special emphasis was placed on the roles of aging, ageism, and social isolation in amplifying these epistemic processes and contributing to delayed recognition of disease, delayed referral, and delayed initiation of treatment.

Findings were synthesized narratively and organized using a conceptual framework grounded in Fricker’s theory of epistemic injustice, explicitly distinguishing between testimonial and hermeneutical dimensions while also examining their interaction [9]. The synthesis prioritized identifying recurring patterns, explanatory mechanisms, and implications for clinical practice, education, and health equity, rather than quantitative aggregation or effect-size estimation. This approach enabled an integrative understanding of how epistemic injustice operates across different stages of RA care.

Reflexivity

The authors’ perspectives as clinicians involved in RA and primary care in aging societies inevitably shaped the interpretive lens of this review. As physicians trained within biomedical frameworks that prioritize objective disease markers, we recognized the possibility of reproducing assumptions that may inadvertently discount patients’ experiential knowledge, particularly in older adults. To address this, we deliberately engaged with literature from multiple disciplines, including medical ethics, social medicine, and qualitative health research, which offer alternative epistemic perspectives on illness experience. During synthesis, we explicitly examined instances where biomedical indicators and patient-reported experiences diverged and critically reflected on how clinical norms regarding credibility, aging, and disease activity might influence interpretation. We also clearly distinguished between empirical findings and conceptual interpretation to maintain transparency in the derivation of conclusions. This reflexive approach aimed to reduce the influence of unexamined biomedical assumptions and to foreground patients’ experiential knowledge as a legitimate source of clinical understanding.

Results

Overview of Included Studies

A total of 18 articles were included in this narrative review following the eligibility criteria described above. The included literature encompassed a broad range of study designs, reflecting the interdisciplinary and conceptually integrative nature of research relevant to epistemic injustice in RA care. These comprised qualitative studies exploring patient experiences and illness narratives; quantitative observational studies examining pain, fatigue, functional impairment, and treatment delay; and narrative reviews and conceptual or ethical analyses addressing patient knowledge, credibility, and clinical interpretation.

The study populations varied across articles but consistently focused on adult patients with RA or closely related chronic inflammatory or musculoskeletal conditions. Several studies specifically addressed older adults or included age-stratified analyses, highlighting age-related differences in symptom recognition, clinical decision-making, and healthcare utilization. Across both qualitative and quantitative studies, persistent pain, fatigue, and functional limitations were frequently reported despite low inflammatory activity or apparently adequate disease control, underscoring the discordance between biomedical disease markers and lived experience.

Thematically, the included articles clustered around four overlapping domains: (i) patient-reported experiences of pain, fatigue, and daily functional burden; (ii) diagnostic and treatment delays in RA; (iii) age-related factors, including ageism and social isolation; and (iv) the interpretation and valuation of patient knowledge within clinical encounters. Although only a minority of studies explicitly used the term “epistemic injustice,” many described phenomena that align closely with its conceptual components, such as the marginalization of patient testimony, the privileging of objective biomarkers over subjective experience, and the absence of shared interpretive frameworks for understanding symptoms in older adults.

Importantly, the included studies originated from diverse healthcare settings and sociocultural contexts, suggesting that these epistemic challenges are not confined to a single healthcare system but represent a broader pattern in RA care. Taken together, the body of literature provides a heterogeneous yet conceptually coherent foundation for examining how testimonial and hermeneutical injustice operate across different stages of RA care and contribute to persistent suffering and treatment delay, particularly in aging populations (Table 1).

**Table 1:** Characteristics of included studies examining patient-reported symptoms and experiences across the RA care trajectory. The included studies span the entire RA care continuum, from symptom onset and time to diagnosis to long-term disease management, highlighting physical, emotional, functional, and social dimensions of living with RA. RA, Rheumatoid arthritis; HAQ, Health Assessment Questionnaire; PGA, Patient Global Assessment; DOT, Dictionary of Occupational Titles (used for work-related functional classification), US Department of Labor [32]

Author(s)	Year	Country	Study design	Stage of RA care addressed	Target population	Key patient-reported symptoms or experiences
Sakalys [15]	1997	United States	Descriptive study	Time to diagnosis	Adult women with probable or definite RA (diagnosed within the prior 2 years)	Pain, fatigue, functional impairment, emotional distress, uncertainty
Hwang et al. [16]	2004	South Korea	Phenomenological study	Post-diagnosis early RA	Adult women with RA (ages 34–61; disease duration 4–12 years)	Severe, uncontrollable pain; fatigue; physical limitation; joint deformity; Emotional distress; Disruption of daily activities, housework, childcare, and social participation
Iaquinta and Larrabee [17]	2004	United States	Phenomenological study+DOT	Long-term illness trajectory	Adults with RA; 6 women (ages 43–67), long disease duration (7–38 years)	Persistent and severe pain, fatigue, stiffness; Functional decline, work disability, early retirement; Emotional distress: grief, fear, anger, frustration, depression; Social withdrawal and concealment of illness
Bergsten et al. [18]	2011	Sweden	Grounded theory	Long-term management	Adults with persistent RA	Pain, fatigue, impaired physical function Unpredictability of disease flares Restrictions in daily activities, and social participation
Barnabe et al. [19]	2014	Canada	Prospective multicenter observational cohort study	Time to diagnosis	Adults with early RA (≤12 months symptom duration)	Patient global assessment of disease activity, Functional impairment measured by HAQ
Östlund et al. [20]	2015	Sweden	Content analysis	Post-diagnosis early RA	Adults with early RA (men and women aged 20–63 years).	Pain, fatigue, stiffness, reduced mobility (especially hands and wrists), tiredness, medication side effects, emotional distress, and altered body image.
Sverker et al. [21]	2015	Sweden	Critical incident technique	Post-diagnosis early RA	Adults aged 20–63 years with early RA receiving contemporary early pharmacological and multidisciplinary care	Pain, fatigue, morning stiffness, reduced strength, functional limitations, and emotional distress
Bala et al. [22]	2016	Sweden	Hermeneutic phenomenological methodology	Long-term disease management	Adults with persistent RA, many older and retired	Persistent, fluctuating pain; Chronic fatigue and morning stiffness; Functional limitations, loss of independence; Emotional distress, frustration, uncertainty, and loneliness
Mølbæk et al. [23]	2016	Denmark	Phenomenological–hermeneutic analysis	Time to diagnosis	Adults recently diagnosed with rheumatoid arthritis (diagnosed within 6 months).	Joint pain, swelling, stiffness, fatigue, diffuse and fluctuating symptoms, functional impairment affecting daily life and work.
Simons et al. [24]	2017	United Kingdom	Qualitative interview study	Time to diagnosis	General public adults (across age groups), without diagnosed inflammatory arthritis	Joint pain, morning stiffness, swelling, uncertainty, and low perceived urgency
Parenti et al. [25]	2020	Italy	Qualitative metasynthesis	Early RA and long-standing RA	Adults living with rheumatoid arthritis (predominantly working-age and older adults).	Chronic and acute pain, stiffness, fatigue, flares, functional impairment, loss of mobility, sexual difficulties, emotional distress, loss of independence, and identity disruption.
Barber et al. [11]	2021	Canada	Focus groups and semi-structured interviews	Entire care trajectory	Multiple stakeholder groups including patients, clinicians, and policy makers	Pain, fatigue, functional limitation; Distress related to long waiting times and delayed access; Need for emotional support, holistic understanding, and continuity
Donnelly et al. [26]	2021	Ireland	Photovoice and thematic analysis	Long-term management	Adults with persistent RA	Chronic pain, fatigue, stiffness, swelling; Cognitive burden; Reduced participation, fear of being a burden, social embarrassment
Pianarosa et al. [27]	2021	Canada	Phenomenological thematic analysis	Entire care continuum	Adults with RA from populations facing inequities: rural and remote residents, Indigenous peoples, elderly persons with frailty, refugees and first-generation immigrants, individuals with low socioeconomic status or unstable housing, and gender/sex-diverse populations.	Delayed diagnosis, difficulty accessing rheumatology care, travel burden, fragmented care, treatment burden, medication access issues, fatigue, multimorbidity, and psychosocial stress.
Stiebitz et al. [28]	2021	Switzerland	Case report	Time to diagnosis	Older adult (81-year-old man) with previously untreated RA	General weakness, desolation, fatigue, weight loss, declining mobility, self-neglect
Ohta and Sano [29]	2023	Japan	Case report	Entire care continuum	Older adults with RA living in rural settings with limited healthcare access	Progressive joint pain, functional decline, fear of disability, financial anxiety, difficulty maintaining daily agricultural activities
Ohta and Sano [30]	2024	Japan	Retrospective cohort study	Time to diagnosis	Adults aged >65 years with newly diagnosed rheumatoid arthritis in a rural Japanese setting.	Joint pain (especially wrist, hand, hip), stiffness, fatigue, systemic pain; symptoms often mild or ambiguous at onset.
Vestergaard et al. [31]	2024	Denmark	Nationwide cross-sectional observational study	Early RA and long-standing RA	Adults (≥18 years) with inflammatory arthritis, including a large subgroup with rheumatoid arthritis	Pain, fatigue, reduced physical function, high Patient Global Assessment, and perceived loneliness

Testimonial Injustice in RA Care

Across the included studies, a consistent pattern emerged in which patients’ reports of pain, fatigue, and functional impairment were devalued when they were not accompanied by clear biomedical indicators of active inflammation. Qualitative studies repeatedly documented that patients experienced persistent and often severe symptoms despite being told that their disease was “stable” or “well controlled,” highlighting a discordance between lived experience and clinical interpretation [16-18,25].

Several studies focusing on early and long-term RA care demonstrated that clinical encounters were strongly oriented toward laboratory findings, imaging results, and clinician-defined assessments, while patients’ subjective experiences were treated as secondary or nonspecific [11,20,21]. When pain or fatigue persisted in the absence of overt inflammatory activity, these symptoms were frequently reframed as psychological distress, general deconditioning, or unrelated comorbidities rather than triggers for reassessment of treatment strategies. This reflects an epistemic hierarchy in which biomedical knowledge is systematically privileged over patient testimony.

The experiential consequences of this credibility deficit were particularly evident in qualitative accounts. Patients described feeling dismissed, not believed, or insufficiently heard when their symptom narratives conflicted with clinical test results [16,17,26]. Repeated encounters in which concerns were minimized eroded trust in healthcare professionals and discouraged patients from openly disclosing symptoms. Some individuals reported internalizing doubts about the legitimacy of their own suffering, illustrating how testimonial injustice can accumulate across clinical interactions over time [17,25].

Importantly, testimonial injustice appeared to be amplified among older adults with RA. Several studies noted that pain, stiffness, fatigue, and functional decline in older patients were more likely to be interpreted as natural consequences of aging rather than manifestations of active or inadequately managed disease [28-30]. Such age-based assumptions further undermine the credibility of patient testimony and intersect with broader patterns of ageism in healthcare. As a result, older adults experience a compounded form of testimonial injustice, in which their accounts are discounted both because objective inflammatory findings are absent and because symptoms are normalized as “age-related.”

Taken together, the included literature indicates that testimonial injustice is embedded within routine clinical reasoning in RA care. By prioritizing biomedical indicators over patients’ experiential knowledge, current practices risk obscuring persistent suffering, discouraging meaningful symptom disclosure, and delaying therapeutic adjustment. These effects appear particularly pronounced in older adults and socially vulnerable populations, contributing to inequities in care and prolonged disease burden.

Hermeneutical Injustice: Aging, Ageism, and Social Isolation

Hermeneutical injustice in RA care arises when patients lack the shared interpretive resources needed to recognize, articulate, and legitimate their symptoms as manifestations of disease. Across the included studies, a recurring theme was the internalization of the narrative that pain, stiffness, fatigue, and functional decline are simply “part of getting older.” This framing was evident not only in clinicians’ interpretations but also in patients’ own accounts, indicating that age-based assumptions were mutually reinforced within clinical and social contexts [28-30].

Several qualitative and case-based studies illustrated how older adults often normalized early RA symptoms, delaying help-seeking because they lacked conceptual tools to distinguish pathological change from expected aging. Patients described uncertainty about whether their symptoms were medically relevant, frequently attributing joint pain or reduced mobility to age-related degeneration rather than inflammatory disease [23,28]. This interpretive gap represents hermeneutical injustice, as individuals are unable to make sense of their experiences in ways that support timely clinical engagement.

Social isolation further intensified these hermeneutical deficits. Studies conducted in rural settings and among older populations highlighted how limited social interaction reduced opportunities for informal comparison, shared storytelling, and collective sense-making around symptoms [29,31]. Without peers or community members to validate their experiences or model help-seeking behavior, patients were less likely to recognize persistent pain or fatigue as abnormal or actionable. In this context, isolation functioned not merely as a social condition but as an epistemic constraint that narrowed the interpretive resources available to patients.

The combined effects of aging narratives and social isolation were closely linked to diagnostic and care-seeking delays. Observational and qualitative studies demonstrated that older adults often postponed initial medical consultation, presented with atypical or advanced symptoms, or experienced prolonged trajectories before referral to rheumatology care [19,23,30]. These delays cannot be explained solely by access barriers; rather, they reflect failures in meaning-making that precede and shape healthcare utilization.

Taken together, the included literature suggests that hermeneutical injustice operates upstream of clinical encounters in RA care, particularly among older and socially isolated patients. When dominant cultural narratives of aging obscure the pathological significance of symptoms and social isolation limits opportunities for collective interpretation, patients are deprived of the epistemic tools necessary to recognize illness and seek care. This hermeneutical gap contributes directly to delayed diagnosis and delayed treatment initiation, reinforcing inequities in RA outcomes in aging societies.

Epistemic Injustice and Treatment Delay

Treatment delay in RA is often framed as a consequence of healthcare system constraints, such as limited access to specialists or prolonged waiting times. However, the included literature suggests that delay frequently originates upstream of institutional barriers, rooted instead in failures of interpretation that shape how symptoms are recognized, communicated, and acted upon. From this perspective, treatment delay can be reconceptualized as an epistemic problem rather than solely a structural one.

Several studies examining the period prior to diagnosis found that patients’ interpretations of early symptoms strongly influenced the timing of their initial medical consultation. Qualitative investigations showed that individuals often experienced diffuse pain, stiffness, or fatigue for extended periods without recognizing these symptoms as warranting medical attention, particularly when symptom onset was gradual or fluctuating [15,23,24]. In these cases, the absence of a shared interpretive framework for early inflammatory disease delayed help-seeking, even before patients encountered formal healthcare barriers.

Once patients entered the healthcare system, epistemic injustice continued to shape clinical trajectories. Studies examining early RA pathways indicated that symptom narratives that did not align neatly with classical inflammatory presentations were often interpreted as low urgency, contributing to prolonged diagnostic intervals [19,24]. This was especially evident among older adults and socially marginalized populations, in whom atypical presentations or multimorbidity complicated clinical interpretation [27,28]. These findings suggest that diagnostic delay is not merely a matter of delayed referral but reflects uncertainty and misalignment in meaning-making between patients and clinicians.

Epistemic injustice also operates after diagnosis, influencing treatment escalation and adjustment. Multiple qualitative studies documented persistent pain, fatigue, and functional limitations despite ongoing pharmacological management, with patients reporting uncertainty about whether these symptoms were legitimate indicators of disease activity or acceptable residual burdens [18,22,26]. When patient-reported experiences were not readily interpretable within dominant biomedical frameworks, they were less likely to prompt therapeutic modification, contributing to prolonged periods of suboptimal symptom control.

These patterns are closely linked to the limitations of treat-to-target strategies when narrowly focused on inflammatory markers and composite indices. While early and aggressive treatment targets have improved objective disease outcomes, several included studies highlighted that patient-reported outcomes (PROs), such as pain, fatigue, and daily functional burden, remained insufficiently integrated into clinical decision-making [11,25]. As a result, patients whose inflammatory activity appeared controlled but who continued to experience significant suffering often found themselves in a therapeutic limbo, where symptoms were acknowledged but not actionable.

Social and contextual factors further amplified these epistemic failures. Studies conducted in rural settings and among socially isolated populations illustrated how limited continuity of care, reduced opportunities for dialogue, and fragmented healthcare encounters constrained shared understanding between patients and clinicians [29,30]. In such contexts, epistemic injustice was reinforced not through overt exclusion but through the cumulative effects of brief consultations, implicit assumptions, and unchallenged narratives about aging, normality, and disease severity.

Taken together, the included literature indicates that treatment delay in RA emerges from the interaction of testimonial and hermeneutical injustice operating across the care continuum, as described in the preceding sections. Hermeneutical injustice limits patients’ ability to recognize and articulate symptoms as medically meaningful, while testimonial injustice diminishes the credibility of those symptoms once expressed. This section synthesizes these mechanisms to illustrate how epistemic injustice operates longitudinally, from symptom interpretation to clinical decision-making. When combined with clinical paradigms that privilege objective indicators over experiential knowledge, these intersecting epistemic processes delay diagnosis, slow treatment escalation, and perpetuate ongoing suffering. Reframing treatment delay as an interpretive failure highlights the need for clinical practices that actively integrate patient testimony and PROs into decision-making, particularly for older adults and socially vulnerable populations.

**Figure 1:**
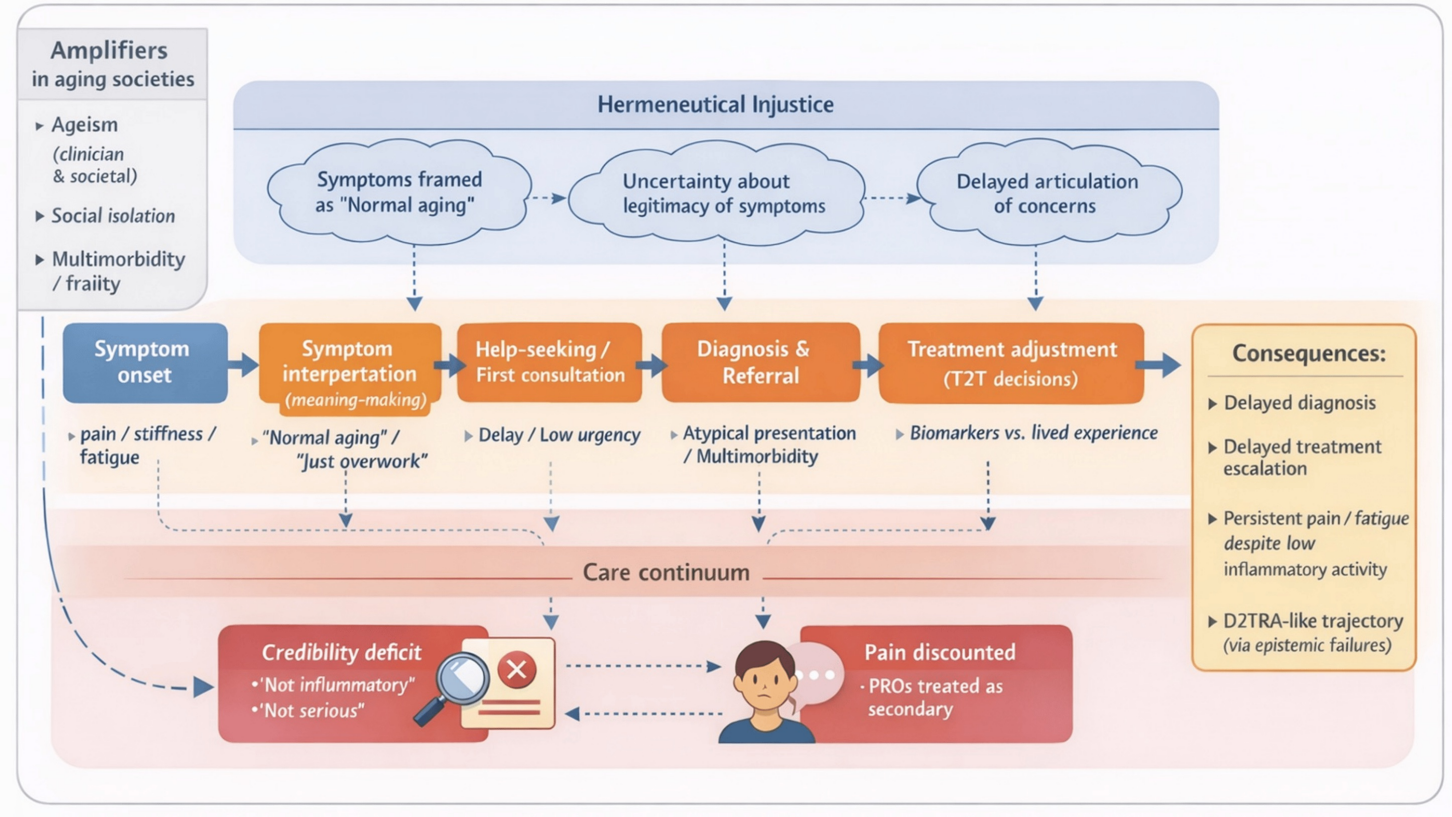
Epistemic injustice across the rheumatoid arthritis care continuum: from symptom onset to treatment adjustment Hermeneutical injustice predominates in the pre-diagnostic phase by constraining symptom interpretation and help-seeking through limited shared interpretive resources, normalization of symptoms as aging-related, and social isolation. Testimonial injustice becomes more prominent after clinical engagement, where patient-reported pain, fatigue, and functional impairment are afforded reduced credibility when objective inflammatory markers appear controlled. Ageism, social isolation, and multimorbidity/frailty act as amplifying factors across stages, contributing to delayed diagnosis, delayed treatment escalation, persistent symptoms despite low inflammatory activity, and D2TRA-like trajectories driven by accumulated epistemic failures. RA, rheumatoid arthritis; T2T, treat-to-target; PROs, patient-reported outcomes; D2TRA, difficult-to-treat rheumatoid arthritis. Image Credit: Ryuichi Ohta

Based on these findings, we developed a conceptual model illustrating how epistemic injustice operates across the RA care continuum (Figure 1). 

Hermeneutical injustice predominates in the pre-diagnostic phase by shaping symptom interpretation and help-seeking behavior, whereas testimonial injustice becomes more prominent after clinical engagement, influencing diagnostic urgency and treatment adjustment.

Discussion

Summary of the Review

This narrative review examined RA care through the lens of epistemic injustice, with particular attention to aging, ageism, social isolation, and treatment delay. By synthesizing qualitative studies, observational research, and case reports, we identified how patients’ experiential knowledge-especially pain, fatigue, and functional decline-was frequently marginalized within routine clinical reasoning. 

Testimonial injustice emerged when patient reports were afforded reduced credibility in the absence of objective inflammatory findings, while hermeneutical injustice operated upstream by limiting patients’ ability to recognize and articulate symptoms as medically meaningful. Importantly, these epistemic failures were not isolated phenomena but interacted across the care continuum, contributing directly to delayed diagnosis, delayed help-seeking, and delayed treatment adjustment. Older adults and socially isolated patients appeared particularly vulnerable to these processes, experiencing compounded forms of epistemic injustice that prolonged suffering despite ongoing engagement with healthcare systems.

Comparison with Other Studies

Previous RA research has often described discordance between patient-reported outcomes and clinician-assessed disease activity, as well as delays in diagnosis and treatment initiation [33-35]. These delays are typically attributed to barriers in healthcare access, system inefficiencies, or patient-level behavioral factors [36,37]. However, such models do not fully explain why delays persist even among patients who actively seek care and remain engaged with healthcare services.

Epistemic injustice provides an additional explanation by highlighting how patients’ experiential knowledge may be undervalued or dismissed during clinical encounters [36,37]. Testimonial injustice may undermine the credibility of patients’ symptom reports, while hermeneutical injustice may limit their ability to express symptoms in clinically recognized terms [34]. These dynamics can contribute to delayed recognition and treatment despite adequate healthcare access, underscoring the importance of more epistemically inclusive clinical practice.

In contrast, the present review extends existing work by conceptualizing delay as an epistemic phenomenon, arising from failures of interpretation rather than solely from institutional constraints. By integrating perspectives from medical ethics and social medicine, this review highlights how dominant biomedical paradigms can obscure persistent suffering when clinical legitimacy is narrowly anchored to laboratory values or composite indices. 

Our findings align with broader discussions in chronic illness literature regarding the marginalization of experiential knowledge but add specificity by demonstrating how aging narratives and social isolation intensify these epistemic dynamics in RA care and other chronic diseases [38-40]. These reframing complements, rather than replace, structural explanations, suggesting that epistemic and institutional factors are mutually reinforcing.

Strengths

A key strength of this review lies in its conceptual integration of heterogeneous literature across different stages of RA care. By including qualitative studies, observational cohorts, and detailed case reports, we were able to trace epistemic injustice longitudinally, from symptom onset and help-seeking behavior to diagnosis and long-term management. 

The explicit use of Fricker’s framework enabled a structured distinction between testimonial and hermeneutical injustice while also examining their interaction in clinical practice. Additionally, the focused attention on older adults and socially isolated populations addresses an important gap in RA research, particularly in aging societies where such patients represent a growing proportion of those affected. Finally, while this review included both empirical studies and conceptual or ethical analyses, empirical studies were prioritised to ensure that ethical and theoretical interpretations were grounded in observed clinical experiences rather than abstract critique alone.

Limitations

Several limitations should be acknowledged. First, as a narrative review, this study does not provide quantitative estimates of the prevalence or magnitude of epistemic injustice in RA care. The heterogeneity of included study designs and populations precludes formal aggregation or causal inference. Second, although the included literature spans multiple countries and healthcare contexts, most studies originate from high-income settings, which may limit transferability to low- and middle-income countries. Third, epistemic injustice was rarely an explicit analytic focus in the original studies; consequently, interpretation required retrospective conceptual mapping, which may introduce interpretive bias. Finally, while this review emphasizes aging and social isolation, other intersecting factors such as gender, ethnicity, and socioeconomic status warrant further dedicated investigation.

Implications for Clinical Practice, Education, and Research

Clinically, addressing epistemic injustice requires more systematic integration of PROs into routine RA management, particularly when inflammatory markers appear controlled [41]. Persistent pain or fatigue should be treated not as residual noise but as epistemically meaningful signals warranting reassessment [35]. In medical education, explicit training is needed to help clinicians recognize credibility biases toward older adults and to reflect on how age-based assumptions shape diagnostic reasoning [8]. Generalist and geriatric perspectives may be particularly valuable in identifying these epistemic blind spots [42,43]. Future research should examine how epistemic injustice contributes to longitudinal disease trajectories, including delayed escalation and persistent symptom burden, using mixed-methods approaches that integrate PROs with clinical outcomes.

## Conclusions

This narrative review suggests that treatment delay in RA cannot be fully understood through structural or biomedical factors alone. Epistemic injustice, manifesting in both testimonial and hermeneutical forms, may significantly influence how symptoms are recognized, interpreted, and acted upon across the care continuum. Clinical practices that prioritize objective indicators over patients’ experiential knowledge, or attribute symptoms to normal aging, may inadvertently contribute to delayed recognition of clinically meaningful disease, particularly among older and socially isolated patients. Reframing treatment delay as, in part, an interpretive and relational challenge highlights the importance of clinical approaches that actively incorporate patient testimony and PROs into diagnostic and therapeutic decision-making. Promoting epistemically inclusive practices, such as attentive listening, validation of patient experiences, and structured integration of patient-reported measures, may represent an important step toward more timely, equitable, and responsive RA care in aging societies.
